# A Gap in Psychiatry On-Call Training: Post-Ligature Assessment

**DOI:** 10.1192/bjo.2022.434

**Published:** 2022-06-20

**Authors:** Maja Donaldson, Ain Nizam

**Affiliations:** Langley Green Hospital, Sussex Partnership Foundation Trust, Crawley, United Kingdom

## Abstract

**Aims:**

1. To assess documented practice on post-ligature assessment following a teaching session and simulated induction session introducing a post-ligature assessment tool. 2. To implement the post-ligature assessment tool, for assessing patients who have tied a ligature, into trust guidance. 3. To support the incorporation of simulated induction teaching on post-ligature assessment into the standard induction timetable delivered to all new trainees in the trust, in order to complete the audit cycle.

**Methods:**

Audit Cycle 1 - Patient data collection November 2020 - January 2021

Action - Locality teaching presenting findings of audit and post-ligature assessment tool developed as part of audit. Concurrent trial of incorporation of post-ligature assessment tool into trust-wide simulation teaching for new trainees.

Audit Cycle 2 - Patient data collection August - October 2021

**Results:**

Audit Cycle 1:

15 incidents

2 involving anchor point/drop

Medic informed in 4 incidents

0 documented in ABCDE format

0 NEWS monitoring

3 follow-up plans documented

3 complications reported

Audit Cycle 2:

10 incidents

0 involving anchor point/drop

Medic informed in 4 incidents

0 documented in ABCDE format

NEWS monitored in 6 incidents

4 follow-up plans documented

3 complications reported

Overall, slight improvement in documentation of NEWS monitoring and follow-up.

**Conclusion:**

Documentation continues to be highly variable. This may be because the teaching done was not trust-wide, simulation session involved only on new doctors in August, some incidents involved locum doctors, and small reach of assessment tool.

We aim to introduce the post-ligature assessment tool as part of trust practice through liaison with the resus teaching team, as well as incorporating it permanently into trust-wide simulation induction teaching.

